# Reducing the size of an alien segment carrying leaf rust and stripe rust resistance in wheat

**DOI:** 10.1186/s12870-020-2306-9

**Published:** 2020-04-09

**Authors:** Sofia Khazan, Anna Minz-Dub, Hanan Sela, Jacob Manisterski, Pnina Ben-Yehuda, Amir Sharon, Eitan Millet

**Affiliations:** Institute for Cereal Crops Improvement, Tel Aviv University, 69978 Tel Aviv, Israel

**Keywords:** Introgression, *Aegilops sharonensis*, SNP, GBS, Rust, Wheat

## Abstract

**Background:**

Leaf and stripe rusts are two major wheat diseases, causing significant yield losses. The preferred way for protecting wheat from rust pathogens is by introgression of rust resistance traits from wheat-related wild species. To avoid genetic drag due to replacement of large wheat chromosomal segments by the alien chromatin, it is necessary to shorten the alien chromosome segment in primary recombinants.

**Results:**

Here we report on shortening of an alien chromosome segment in wheat that carries leaf and stripe rust resistance from Sharon goatgrass *(Aegilops sharonensis)*. Rust resistant wheat introgression lines were selected and the alien region was mapped using genotyping by sequencing. Single polymorphic nucleotides (SNP) were identified and used to generate diagnostic PCR markers. Shortening of the alien fragment was achieved by induced homoeologous pairing and lines with shortened alien chromosome were identified using the PCR markers. Further reduction of the segment was achieved in tertiary recombinants without losing the rust resistance.

**Conclusions:**

Alien chromatin in wheat with novel rust resistance genes was characterized by SNP markers and shortened by homoeologous recombination to avoid deleterious traits. The resulting wheat lines are resistant to highly virulent races of leaf and stripe rust pathogens and can be used as both resistant wheat in the field and source for gene transfer to other wheat lines/species.

## Background

Leaf and stripe rusts are two major wheat diseases that cause tremendous annual losses in wheat worldwide. For example, from 2000 to 2004 in the USA, losses to leaf rust were estimated at over 3 million tons, worth over $350 million [1] and losses due to stripe rust in 2003 were 3.4% or 11.7 million tons [2]. The incidence of severe rust outbreaks has intensified in the past years; primarily due to the appearance of highly virulent races that overcome wide spread rust resistance sources [3, 4]. This situation is expected to worsen due to climatic changes that create favorable conditions for disease outbreaks [5]. Since the high rate of pathogen virulence alterations quickly erodes the primary pool of rust resistance genes, new sources of rust resistance are needed to boost this gene pool of wheat.

Breeding for rust resistance is the most economically and environmentally safe way to control rust diseases. The conventional method used since the 1920s to boost the wheat gene pool is introgression of new rust resistance genes from wheat-related wild species [6–8]. To date, more than 70 resistance genes against leaf rust and stripe rust originating in wheat or in its wild relatives have been identified and mapped [9]. Most of these genes confer seedling resistance although adult plant resistance (APR) genes were also identified [10, 11].

Despite being a rich gene pool, only few of the resistance genes were introgresed from *Aegilops* spp., and especially from species of section Sitopsis (diploids with S or modified S genomes). Exceptional is *Aegilops speltoides* that provided 6 leaf rust resistance genes *Lr28, 35, 36 47 51 and 6*6 [9]. *Aegilops sharonensis* Eig (Sharon goatgrass) was found to possess resistance against different wheat diseases including leaf, stripe and stem rusts, powdery mildew, tan spot and spot blotch [12] but has not been widely exploited [13]. In 2006, Marais et al. reported on transferring resistance to leaf rust (*Lr56*) and stripe rust (*Yr38*) to chromosome 6A of wheat, while recently, Millet et al. [14] reported on introgressing resistance to the devastating stem rust race TTKSK (Ug99) from *Ae. sharonensis* to bread wheat.

One of the main challenges faced by the breeders is restricted recombination between homoeologous chromosomes, which is necessary to transferring the resistance from related species [15]. Usually, in hexaploid wheat chromosome pairing is allowed between homologous chromosomes while suppressed between homoeologous chromosomes by the *Ph1*, localized on the long arm of chromosome 5B [16, 17]. To allow for homoeologous pairing, deletion lines of the *Ph1* locus (*ph1b*) were developed to induce pairing and recombination between homoeologous chromosomes when their homologues are absent [18]. Currently, *ph1b* is extensively used for transferring various resistance genes from the wild relatives to wheat [19].

Classically, cytogenetic analyses, such as fluorescent in situ hybridization (FISH) and genomic in situ hybridization (GISH), have been used for the detection of introgressions. These techniques are insufficiently precise to identify the translocation breakpoints [20] and require expertise which is not a common knowledge [21]. In addition, molecular markers such as DArT and microsatellites, that have been previously used to characterize the *Ae. sharonensis* chromatin [22–24], were too sparsely distributed to resolve the precise translocation. Moreover, the advances in sequencing technologies and the availability of a wheat reference genome [25], allowed for a more precise characterization of the alien segment [26]. Genotyping-by-sequencing (GBS) is one of the leading marker identification tools, based on reducing the genome complexity by using restriction enzymes [27]. Using GBS it is possible to isolate large number of SNPs for cost-efficient high density genetic mapping and development of markers for a high-throughput identification of the translocated segment [28–30].

In a previous work, a segment carrying resistance gene(s) to both leaf and stripe rust of wheat was introgressed from *Ae. sharonensis* into chromosome 6B of bread wheat [23]. A number of wheat lines were generated with recombinant 6B chromosome carrying *Ae. sharonensis* 6S^sh^ chromosome segment of different lengths (ranging from most of the chromosome to ~ 20 cM). The introgression lines showed complete resistance against a broad range of leaf and stripe rust races. However, they all possessed a chromosomal segment that is large enough to potentially affect agricultural and product desired traits, since several important genes and gene families span all over chromosome 6B. Such genes include gliadins, glutenins, grain protein content gene (*GPC B1*), restorer of fertility (*RF 4*) and supernumerary spikelet (*SS*) [31, 32]. The introduced segment of *Ae. sharonensis* might have replaced these genes, hence to avoid deleterious effect on grain and yield, the cultivated wheat genetic background should be largely restored.

The objectives of present work were to reduce the size of the introgressed segment, by derivation of secondary and tertiary recombinants, without losing the novel rust resistance, and to develop SNP-based markers for the identification of the alien segment. The new recombinants will provide a new rust resistance resource for breeding durable cultivars. The developed PCR markers will be helpful for selection of resistant progeny by breeders.

## Results

### Production and molecular characterization of plants with reduced size alien introgression

**Fig. 1:**
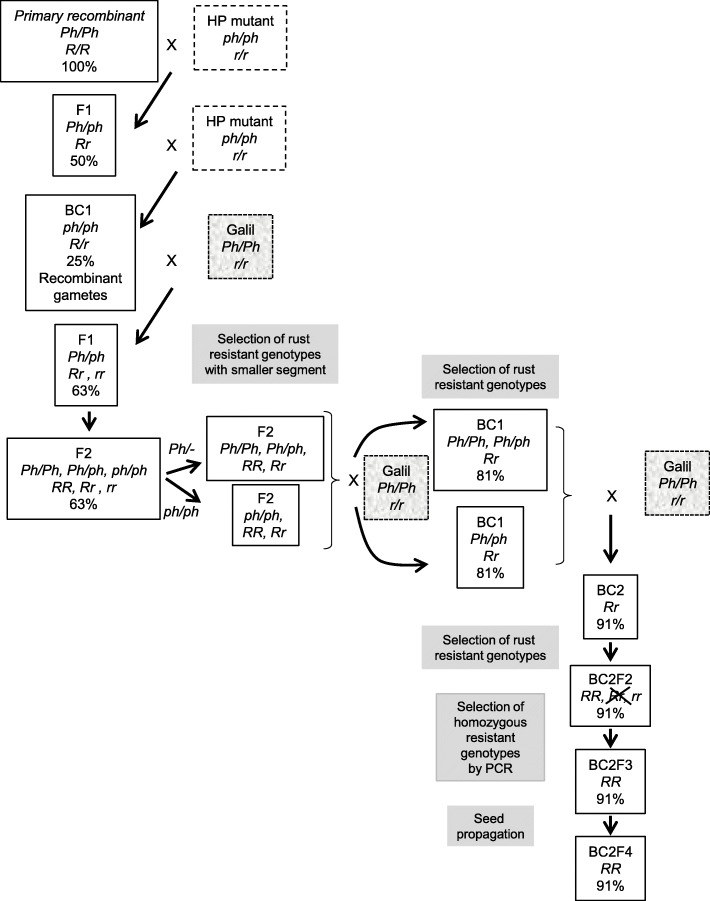
Procedure for the derivation of secondary and tertiary recombinants. The procedure of the alien segment shortening is described in the Methods section. R and r denote for presence or absence of the alien resistance gene, respectively. *Ph* and *ph* denote for *Ph1* and *ph1b* alleles, respectively. Percentage figures are calculated rate of cv. Galil chromatin

**Fig. 2:**
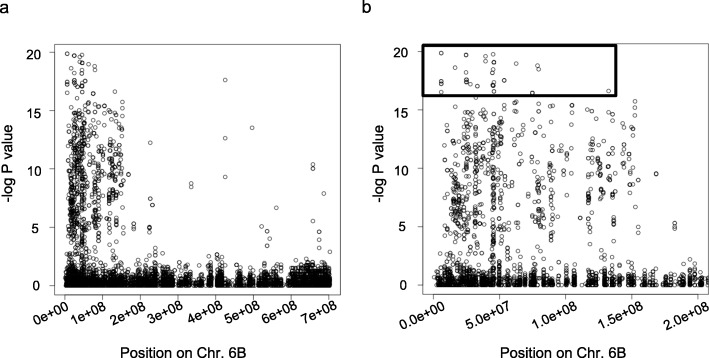
Statistical analysis of chromosome 6B SNPs. –log *P*-value of Fisher Exact test conducted on SNPs between *Ae. sharonensis* and bread wheat in chromosome 6B (based on alignment to CS genome). X-axis represents the position of each SNP (represented in circles) on CS genome, Y-axis is the –log *P*-value of Fisher Exact test. **a** Total number of SNPs. **b** Zooming into the area of potential SNPs. SNPs with –log *P* > 16 are boxed

**Fig. 3:**
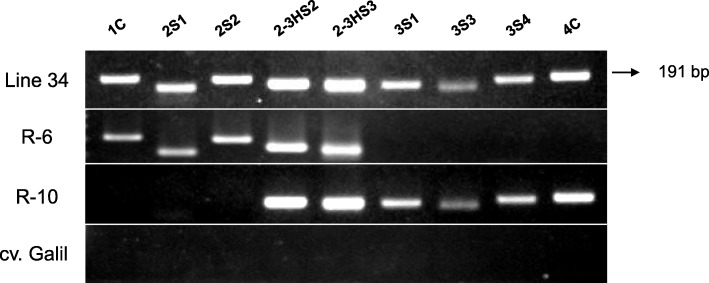
Gel electrophoresis representing all of the PCR markers for assessment of the segment boundaries. cv. Galil is the susceptible elite cultivar (lack of bands represents absence of the *Ae. sharonensis* segment). Line 34 is one of the primary recombinants that served as a positive control. R-6 is an example to a secondary recombinant with a shortened segment towards the long arm telomere, R-10 is an example to a secondary recombinant that recombined towards the short arm telomere. Four Gel images were cropped and combined together for clarity

**Fig. 4:**
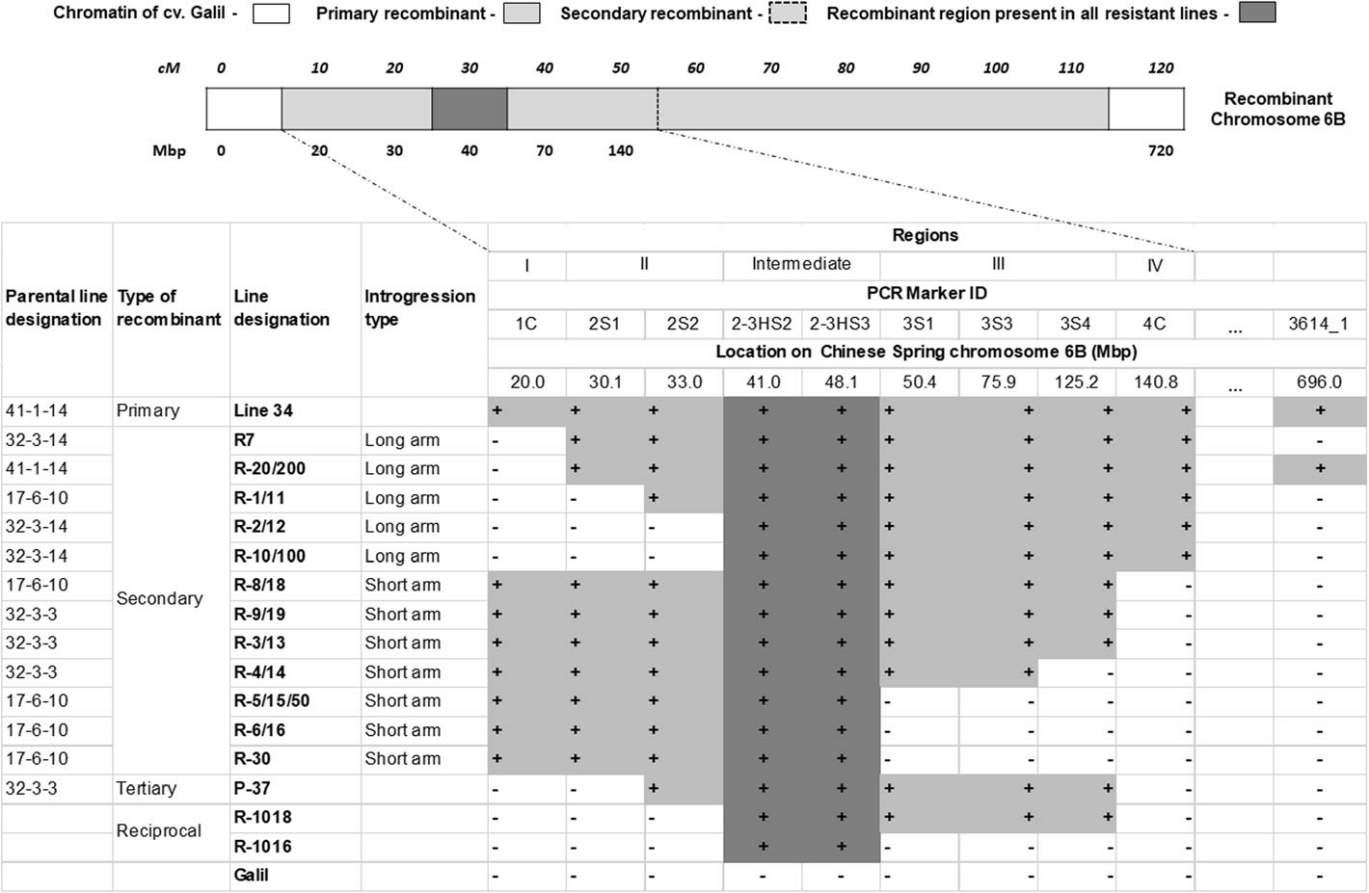
Chromosome 6B constitution. Presented according to [23] and the analysis with PCR markers 1–9 (Table 1). Wheat lines presented include primary recombinant line 34, 12 secondary recombinant lines (R-), one tertiary recombinant line (P-37), R-1018 line that was derived from the cross of R-10 and R-18, and R-1016 line that was derived from the cross of R-10 and R-16. cv. Galil is the susceptible elite cultivar without the *Ae. sharonensis* segment. A segment spanning 0–140 Mb of recombinant chromosome 6B was divided into four regions (I-IV) restricted by markers (1C-4C). Symbols + and - indicate presence or absence of the *Ae. sharonensis* markers, respectively. Light grey and white colors represent presence or absence of *Ae. sharonensis* segment, respectively. The boxed (intermediate, dark grey color) region is the alien region present in all of the resistant recombinants. Regions I and II are left extensions (towards the short arm telomere); Regions III and IV are right extensions (towards the long arm telomere)

**Fig. 5:**
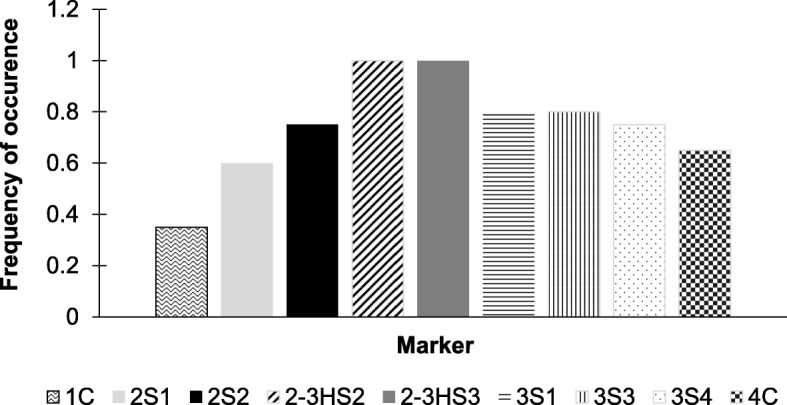
Frequency of occurrence of the PCR markers for assessment of the segment. Frequencies are calculated from 20 candidates that were screened with all of the markers

A population of 1240 plants, derived from the first cross of the *ph1b*/*ph1b* BC_1_ with cv. Galil (Fig. 1), were inoculated with the leaf and stripe rust isolates. A total of 594 plants were found resistant to both isolates, 45 were found resistant to one of the isolates (28 to leaf rust and 17 to stripe rust), and the other 601 plants were susceptible to both isolates. GBS analysis of 100 selected plants revealed SNPs scattered along the homoeologous recombination region on chromosome 6B. In order to define more precisely the location of the alien segment in specific introgression lines, the sequences of the recombinants were re-aligned to the sequence of the bread wheat cv. Chinese Spring (CS) [33]. Fisher Exact test was conducted to discover SNPs associated with resistance. Totally, 1362 SNPs were found, most of which were located in the range of ~ 0–160 Mbp (Fig. 2a). Twenty-six of these SNPs had *P* values of -logP> 16 (Fig. 2b) and were most capable to distinguish between the chromatin of *Ae. sharonensis* and cv. Galil. The recombined area was further divided into four regions, according to the frequency of recombination as reflected by the SNPs. Finally, we designed PCR markers from seven SNPs that span the four regions of the entire segment (Table 1), and used them to screen the remaining 520 plants that were found resistant to both isolates (Fig. 3). At first, all of the plants were screened with the two distal markers 1C and 4C. Presence of both markers indicated lack of recombination within the alien segment, absence of one of the markers indicated recombination on either the left side (lack of marker 1C), or the right side (lack of marker 4C) of the alien segment. We identified 38 plants that were deficient for one of the markers and these plants were further evaluated using the remaining five PCR markers. Based on the PCR analyses we selected a set of 20 secondary recombinant (SR) plants. These plants were re-evaluated for the resistance and screened again with the PCR markers. From these plants, we selected a final set of 13 recombinant lines based on the length of the alien fragment, the introgression pattern (Fig. 4), and on their phenotypic resemblance to cv. Galil. All of these lines contained a common region of 17.4 Mb that was detected between markers 2S2 and 3S1, regardless if the break point of the recombination occurred at the right or the left end. To verify this region, we developed two additional markers, 2-3HS2 and 2-3HS3 (No. 8–9 in Table 1), and used them to analyze the selected recombinants as well as randomly selected susceptible lines, which were used as a negative control. Both markers were present in all of the resistant recombinants (Fig. 4) and absent in the susceptible lines (not shown). The frequency of occurrence of each marker was calculated to indicate the rate of recombination along the chromosome segment (Fig. 5). Markers 2-3HS2 and 2-3HS3 were both present in all resistant plants, 3S1 and 3S3 were each present in 16 plants, 2S2 and 3S4 were each present in 15 plants, 4C in 13 plants, 2S1 in 12 plants, and 1C was present in seven plants. In total, 2.5% (13/520) and 1.34% (7/520) of the resistant plants had a secondary recombination on the left end (indicated by lack of marker 1C) or the right end (indicated by lack of marker 4C) of the introgression segment, respectively.

**Table 1:** PCR markers for mapping the *Ae. sharonensis* alien segment

No.	Marker name	Primer (5′-3′)		Annealing Temp^a^ (C°)	Marker size (bp)
		Forward	Reverse		
1	1C	GGCCAGTGCAATAAACT	ATTTGTAGTAAGAGTGC**G**	56	214
2	2S1	AAAAGAAAGTTGGCCCC**G**	CGGCATGATTAAAACATGAGGCA**A**	61	128
3	2S2	TCATCGA**T**GG**G**ATCGA**C**	ATGTCCACCTGTCCCAAG	62	209
4	3S1	ATCCTATCGCTCAACAT**G**	ACAGTTAGCTTGGCTTC**A**	56	147
5	3S3	TGATGGATTGGATTAAAAACTTG	GTCCTTTTCTCCATCTTCAT**C**	56	153
6	3S4	GCTGCGTAAAATTAAGCA	CTTTTAGTCAA**T**TCTTGGT**C**	56	180
7	4C	CTCAATCATTTCCGTCTAC	CTACGCAACAAGGAAAAC**A**	57	191
8	2-3HS2	CAAACACCACAACAGCTATG	CAGCCCGAAGGAAAACAA**T**	61	154
9	2-3HS3	CAATTGGCATAAGAGCCT**T**	CTCGACGATGATGAAGAC	61	144
10	PSR2120	TTAACGCCAGGGCATACTC	CTGCAGGAGGCGCTGGA	58	232
11	Zyg_1Sh_1	CAAACACCACAACAGCTATG	CAGCCCGAAGGAAAACAA**T**	61	154
12	Zyg_2G_2	CAATTGGCATAAGAGCCT**G**	CATAGCCATCACCACCTTG	58	219
13	3614_1	ATCGCAAGGTGTTGTCCATT	GGCA**G**CTGGAAGATCAA**G**TC	56	165

In all markers, which are based on SNPs, the polymorphisms are in bold. Markers 1–7 were used to characterize the length of the segment. Markers 8–9 were used for detection of the critical area for the resistance. Marker 10 was used for selection of *ph1b/ph1b* lines. Markers 11–12 were used simultaneously for selection of plants homozygous for the segment (plants in which marker 11 was present and marker 12 was absent were selected). Marker 13 was used for the assessment of recombinant area at the distal long arm telomere area. Temperature cycling consisted of 95 °C for 5 min, followed by 32 cycles of 95 °C for 30 sec, Annealing Temperature (see table for details) for 30 sec, 72 °C for 30 sec with a final extension step at 72 °C for 5 min

^a^Annealing temperature varied depending on the Taq polymerase used

The final set of 13 selected SR lines were allowed to self-pollinate and between 20 and 30 F_2_ offspring of each SR line were evaluated for their reaction to the leaf and stripe rust isolates. PCR analysis of the resistant F_2_ plants with *Ph1* specific marker revealed 12 lines that contained the *Ph1* allele and one line lacking it. The 12 *Ph1*-positive lines had different alien chromatin constitution: in seven lines the alien segment extended towards the short arm telomere, while in the remaining five lines the alien segment extended towards the long arm telomere (Fig. 4). All of these SR 6B plants were backcrossed to cv. Galil and rust resistant (R/r) progenies were selected. In this cross, the single genotype that was found homozygous for *ph1b* underwent another homoeologous pairing event and produced a tertiary 6B recombinant (TR) chromosome in line P-37 (Fig. 4). All of the 12 SR lines and the single TR line were further backcrossed (BC_2_) to cv. Galil (Fig. 1) in order to recover the genetic background.

### Further shortening of the segment

To further reduce the size of the alien segment we performed a cross between two SR BC_2_ plants, one with a right arm tail (Figure S1, type a) and one with a left arm tail (Figure S1, type b). The progeny of this cross was analyzed by PCR using the diagnostic markers (Table 1) and two hybrids were selected. One hybrid (R-1018) contained an alien segment that was derived from chromosome 6B in SR lines R-18 and R-10 and contained the 2-3HS2 and 2-3HS3 markers (Fig. 4). The alien segment in this line was limited by markers 2-3HS2 and 3S4, indicating even a shorter segment than in the TR line P-37 (Fig. 4). The other hybrid (R-1610) contained an alien segment that was derived from chromosome 6B in SR lines R-16 and R-10 and contained the 2-3HS2 and 2-3HS3 markers but also was limited by these markers. This hybrid possessed the shortest segment of all the introgression lines, however, self-pollinated progeny of 6B heterozygous chromosome (recombinant and Galil) from crosses with cv. Galil, were unexpectedly all heterozygote for 6B.

### Marker assisted selection of homozygous plants for advanced backcrosses

Homozygous resistant plants were selected from self-pollinated BC_2_ progeny by their alien segment status as follows. At least 100 BC_2_F_2_ plants from each SR line were screened with the PCR markers Zyg_1Sh_1 and Zyg_2G_2 (Table 1). Self-fertile lines with cv. Galil morphology, that had the desired alien chromatin (Zyg_1Sh_1 - positive) and lacked the cv. Galil PCR marker Zyg_2G_2, were selected. BC_2_F_3_ seeds of the selected homozygotes of each SR line were pooled and used to produce BC_2_F_4_ seeds for further experiments, including resistance to stripe rust in the field.

### Adult plant reaction of the secondary and tertiary recombinant lines

#### Reaction to leaf rust

The reaction of the recombinant lines is presented in Table 2 as percentage of uredinia coverage and infection type value. A score of 3 was considered susceptible, whereas 0 to 2 reflected descending values of resistance. All the recombinant lines were highly resistant except line R-4 which segregated into highly resistant or susceptible individuals. Wheat cv. Galil had more than 80% coverage and was scored 3 IT value, hence considered as a susceptible genotype.

**Table 2:** Adult plant reaction of the recombinant lines to inoculation by leaf rust isolate #526–24 in the greenhouse

Line		Flag leaf		-3 leaf		General reaction^b^
		% coverage	IT value^a^	% coverage	IT value^a^	
R-1(−2–103)		7.4	0; to 2	26.0	0; to 1+	R
R-1(−2–104)		3.4	0; to 1	19.1	0; to 1- (2)	R
R-2		25.0	0; to 2 (3)	32.2	0; to 2 (3)	MR
R-3		2.8	0; to 1+	14.3	0; to 1+	R
R-4	R	7.0	0; to 1-	20.0	0; to 1+	R
	S	70.0	3	80.0	3	S
R-7		10.0	0; to 1+	24.5	0; to 1	R
R-10		1.8	0; to 1	20.6	0; to 1-	R
R-16		6.2	0; to 1	21.0	0; to 1 (2)	R
R-18		1.8	0; to 1	10.1	0; to 1	R
R-19		6.7	0; to 2	16.9	0; to 1+	R
R-20		8.2	0; to 1 (2)	18.9	0; to 1=	R
P-37		0.8	0; to 1	16.6	0; to 1-	R
Line-33		2.3	0; to 1=	14.1	0; to 1-	R
Line-34		3.4	0; to 1	10.6	0; to 1	R
Line-42		3.4	0; to 1-	16.3	0; to 1	R
cv. Galil		81.7	3 (2)	63.8	3	S

Line R-4 segregated into half resistant (R) and half susceptible (S) plants

^a^Values are mean of 10 plants, IT values in brackets denote rare scores on the same leaf

^b^General reactions were as follows: 0 to 0;: highly resistant (HR); 0;1 to 1: resistant (R); 1 to 2: moderately resistant (MR); 2 to 3−: moderately susceptible (MS); and 3− to 4: susceptible (S)

#### Reaction to stripe rust

**Fig. 6:**
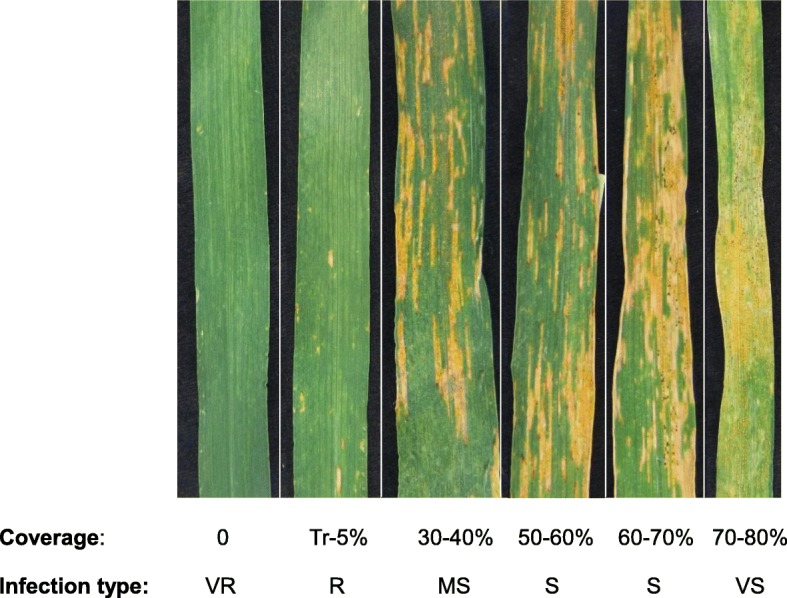
Scale of reaction to field inoculation by spreader of stripe rust isolate #5006. ^*^VR = very resistant; R = resistant; MS = medium susceptible; S = susceptible; VS = very susceptible

All the recombinant lines were highly resistant (VR) with 0 coverage (R-1-2-103 and R-1-2-104 gained 0-traces) (Fig. 6) except line R-4, which segregated into 0 (VR) and 50–60% coverage (S). cv. Galil scored 30–40% (MS to S) and Falchetto which was planted in every plot, scored 60–70% (S) or 70–80% (VS).

### Distorted segregation of the *Ae. sharonensis* chromatin

Distorted segregation was observed in most of the lines in all generations (Tables 3 and 4), and the rate of homozygous resistant plants was usually lower than the expected 25%. Moreover, resistant plants of the SR lines R-5, R-6 and R-30 yielded none or almost no homozygous plant for the alien segment and were not propagated to advanced generations. These lines typically lacked alien chromatin in regions III and IV (Fig. 4), that was present in the rest of the SR lines.

**Table 3:** Segregation of BC_2_F_2_ progeny of heterozygous alien translocation plants into rust resistant and homozygous rust resistant plants

Line	No. of BC_2_ plants	No. of BC_2_F_2_ progeny plants	Heterozygous Resistant			Homozygous Resistant			Χ^2^ *
			No. of plants		% of plants	No. of plants		% of plants	
			Observed	Expected		Expected	Observed		
R-1	3	257	152	128.5	59.1	39	64.25	15.2	14.27
R-2	2	190	113	95	59.5	17	47.5	8.9	26.28
R-3	2	215	128	107.5	59.5	28	53.75	13.0	16.76
R-4	2	188	107	94	56.9	10	47	5.3	43.18
R-5	3	274	173	137	63.1	0	68.5	0	93.38
R-6	4	396	255	198	64.4	2	99	0.5	127.61
R-7	1	95	47	47.5	49.4	12	23.75	12.6	12.14
R-8	3	285	187	142.5	65.6	45	71.25	15.7	28.24
R-9	2	197	111	98.5	56.3	24	49.25	12.2	17.83
R-10	2	190	115	95	60.5	34	47.5	17.8	8.94
R-20	2	188	134	94	71.3	21	47	11.1	35.57
R-30	2	155	79	77.5	51.0	1	38.75	0.6	70.72

Resistance to leaf rust pathogen was determined at seedling stage. PCR marker Zyg_1Sh_1 was used to determine presence of *Ae. sharonensis* resistance segment, markers Zyg_1Sh_1 and Zyg_2G_2 determined zygosity of the plants. Expected values are 50% heterozygous, 25% homozygous resistant and 25% homozygous susceptible plants

*All Χ^2^ values present significant (*p* > 0.05) deviation from the expected distribution

**Table 4:** Segregation of 6B-chromotypes in BC_3_ and BC_4_ populations

Line	BC_3_F_2_ population					BC_4_F_2_ population				
	Total No. of plants	No. of non- recombinants	No. of heterozygotes	No. of recombinant homozygotes^a^	Χ^2^ *	Total No. of plants	No. of non- recombinants	No. of heterozygotes	No. of recombinant homozygotes^a^	Χ^2^ *
**R-1**	48	15	25	8 (12)	2.13	39	5	28	6 (9.75)	7.46*
**R-2**	33	11	18	4 (8.25)	3.24	23	8	13	2 (5.75)	3.52
**R-3**	33	9	20	4 (8.25)	3.00	26	7	17	2 (6.5)	4.38
**R-4**	33	16	16	1 (8.25)	13.67*	25	8	13	4 (6.25)	1.32
**R-16**	33	11	22	0 (8.25)	11.00*	19	5	14	0 (4.75)	6.89*
**R-7**	50	10	28	12 (12.5)	0.88	26	4	16	6 (6.5)	1.69
**R-18**	34	7	16	11 (8.5)	1.06	25	10	15	0 (6.25)	9.00*
**R-19**	33	14	16	3 (8.25)	7.36*	32	7	17	8 (8)	0.19
**R-10**	33	12	9	12 (8.25)	6.82*	25	7	7	11(6.25)	6.12*
**R-20**	33	10	17	6 (8.25)	1.00	25	9	14	2 (6.25)	4.28
**P-37**	31	8	19	4 (7.75)	2.61	25	6	13	6 (6.25)	0.04

PCR marker Zyg_1Sh_1 was used to determine presence of *Ae. sharonensis* resistance segment, markers Zyg_1Sh_1 and Zyg_2G_2 determined zygosity of the plants. BC_4_ was produced by pollinating heterozygous BC_3_ by cv. Galil

*Significant (*p* > 0.05) deviation from the expected distribution

^a^Values in brackets denote the expected number of recombinant homozygotes

To evaluate female and male transmission, line R-1610, which yielded in self-pollination a progeny of only heterozygotes for 6B, was reciprocally crossed with cv. Galil. The F_1_ plants were molecularly analyzed and showed refrained female transmission of recombinant marker Zyg_1Sh_1 (Table 5), while the male transmission exceeded 64% rather than the expected 50%.

**Table 5:** Male and female transmission of the recombinant *Ae. sharonensis* Zyg_1sh_1 marker in heterozygous F_1_ plants of reciprocal crosses

Cross	No. of F_1_ progeny plants	No. of plants with *Ae. sharonensis* chromatin	% of plants
R-1610 X cv. Galil	48	0	0
cv. Galil X R-1610	45	29	64.4

Heterozygous R-1610 plants were reciprocally crossed with cv. Galil. PCR marker Zyg_1Sh_1 was used to determine presence of *Ae. sharonensis* resistance segment

## Discussion

Wild plant species contain many desirable traits that can be used to improve related crop plants. However, transfer of traits from wild plant species to related crops by introgression results in replacement of large chromosome segments of the crop plant by an alien chromosomal segment and often leads to reduced yield, a phenomenon known as genetic drag. Following the initial introgression, it is therefore necessary to reduce the size of the introgression segment to minimize unwanted side effects. This task is especially challenging in wheat due to genetic mechanisms that prevent pairing of homoeologous chromosomes. As a result, many of the alien genes that were transferred into wheat by chromosome engineering have not been used due to the associated yield penalty [7, 34–37].

Here, we used *ph1b*-induced homoeologous pairing to reduce the size of an alien segment carrying rust resistant genes that were introgressed into wheat from Sharon goatgrass. Our crossing procedure allowed us not only to reduce the primary segment by induction of recombination between the wheat DNA and the original alien segment, but also to further trim it by repeated backcrosses with the parental wheat line and selection of homozygous *ph1b* plants at each backcross cycle. A further shortening on both sides of the segment was achieved by hybridizing two types of SR lines that paired and recombined in their overlapping region. Phenotypic screening accompanied by marker-assisted selection (MAS) facilitated the initial and further shortening of the introgression.

Different techniques were developed to enable the detection of *Aegilops* spp. chromatin in a wheat genetic background [20]. C-banding was used mainly to detect whole chromosome addition and substitutions. FISH and GISH were used to detect alien translocations but their resolution ability is not adequate to point precisely on the translocation breakpoints. The fact that *Aegilops* and *Triticum* spp. both have a common ancestor hampers the detection ability of substitution of *Aegilops* segment for its wheat homoeologue. Molecular approaches such as DArT [23, 24] or SSR [22] that were used to detect *Ae. sharonensis* chromatin were not satisfactory for a precise characterization of the introgression due to sparse or unequivocal markers. Hence, GBS was performed and confirmed the mapping of the alien segment to the 6B chromosome by the previous DArT analysis [23]. Subsequent development and utilization of SNP-based molecular markers (Table 1, 1–9) assisted analysis of the introgression boundaries and selection of desired shortened secondary and tertiary recombinant lines. In particular, a ~ 17 Mbp region, confined by markers 2-3HS2 and 2-3HS3, was identified as necessary for the resistance as it was present in all the resistant lines and absent from the susceptible (Fig. 4). Additional dominant molecular markers allowed for phenotypic selection of homozygous plants avoiding unnecessary round of phenotypic selection (Table 1, 11–12). Since the markers are located within the alien segment and are associated with the resistance, they may also be used in future breeding programs for detection of the resistance genes. We conclude that the combination of induced homoeologous recombination by *ph1b*, together with crosses of non-identical SR lines, in combination with molecular analysis, considerably fosters shortening of an alien segment in wheat.

To allow homeologous pairing, the rust resistant *Ae. sharonensis* line was crossed with wheat cv. CS *ph1b* mutant line [23]. The rust resistance was then transferred to a commercial wheat line by crossing the primary cv. CS introgression lines with the elite line cv. Galil (Fig. 1). Therefore, the BC_2_ generation of the SR and TR lines contains about 10% of cv. CS, which needs to be further reduced by additional backcrosses to cv. Galil prior to evaluation of the field performance of the SR and TR lines. For example, during the course of the study, we noticed that some of the introgression lines suffered from leaf chlorosis. While hybrid necrosis or chlorosis is frequent in tetraploid by hexaploid wheat hybrids, due to the action of two complementary genes [38, 39], chlorosis was not present in earlier generations in the greenhouse and emerged in field plots after three crosses to cv. Galil. Although the reason for the chlorosis remains unclear, we expect that this defect is associated with the remaining CS chromatin and that it will be eliminated in more advanced backcross generations.

Another observation indicated that in some cases the genetic segregation of the alien segment was distorted. Distorted segregation is a widespread phenomenon in plant species including wheat and its relatives [40, 41]. Distorted segregation can be observed as quantitative deviation from Mendelian segregation but can reach a total exclusion of a certain type of gametes from fertilization as in the case of pollen killer (*Ki*) [42] and gametocidal (*Gc*) genes [43]. Indeed, we observed a general reduction in transmission of gametes with recombinant 6B chromosome compared to expected theoretical segregation (Tables 3 and 4). The extreme deviation from the expected ratio of homozygous resistant BC_2_F_2_ progeny plants was observed in the SR lines R-5, R-6, and R-30 (Table 3). These lines have a similar constitution of the alien chromatin towards the short arm telomere and are limited by the 2-3HS3 marker (Fig. 4). In contrast, the SR line R-4, in which segregation was not so severely distorted, also contains a fragment that extends towards the short arm telomere, however in this line the segment is slightly longer as it includes marker 3S3 (Fig. 4). It is possible that an unknown factor of the wheat chromatin resides between markers 2-3HS3 and 3S4 (located between 48.1 and 125.2 Mbp on chromosome 6B) and prevents transmission of the gametes by interaction with the *Ae. sharonensis* chromatin on the short recombinant 6B chromosome arm. Moreover, the recombinant 6B chromosome was not transmitted at all through the female gametes, in the reciprocal crosses of the shortened hybrid line R-1610 and cv. Galil, resulting in no F_1_ progeny with *Ae. sharonensis* resistance segment (Table 5). Marais et al. [44] however reported on differential transmission of wheat and recombinant 6A chromosome with translocation from *Ae. sharonensis*, where exclusive (100%) male transmission and reduced (35%) female transmission of the recombinant chromosome was evident. The hypothesis that this phenomenon stems from the action of the pollen killer gene, that might be present on chromosome 6B [45] of cv. Galil but not on the recombinant chromosome that contains chromatin of cv. CS and *Ae. sharonensis* was rejected in our case, since male transmission was not negatively affected (Table 5). The involvement of a gametocidal gene was also rejected since it is active on both male and female gametes and should bring about a half seed set in a self-pollinating spike, which was not the case here. While the mechanism(s) behind the reduced transmission rates of the alien chromosome remain to be found, it is important to note that reduced gamete transmission is expressed only in heterozygotes and therefore it does not pose any constraints on grain production of a homozygous plant. Indeed, plants that are homozygous for the recombinant gave rise to fully fertile spikes.

## Conclusions

We demonstrated the usefulness of the *ph1b* mutation for induction of recombination between wheat and *Ae. sharonensis* chromatin, and established a working protocol for repeated trimming of an alien segment, accompanied by marker-assisted selection. Decrease in the segment size was achieved first through homoeologous recombination in background of a *ph 1* mutant, and then by homologous recombination of two different types of recombinants. The procedure allowed for reduction of the introgressed segment together with potential negative effects on grain yield and quality, without harming the rust resistance.

We also showed here the efficiency of the GBS method in the identification of SNPs that can be used for efficient selection of the resistant recombinants in future breeding programs.

## Methods

In this study, we aimed to reduce the size of the introgressed alien segment from rust resistant primary recombinants. The primary recombinant lines were produced using *ph1b* induction of homoeologous pairing between bread wheat cv. Chinese Spring (CS) and Sharon goatgrass chromosomes followed by backcrossing to the recurrent wheat parent cv. Galil [23]. Here, to reduce the size of the alien segment, we produced secondary and tertiary recombinants using the *ph1b* mutant, without losing the novel rust resistance.

### Plant material

The recombinant lines that were selected for this work were produced by E. Millet [23] and their seeds are deposited in the Harold and Adele Lieberman Germplasm Bank at Tel Aviv University; their 6B chromosome constitution is presented in Table 6. The homoeologous pairing *ph1b* mutant (HP) in the genetic background of cv. CS was originally obtained personally from the late E.R. Sears. Wheat cv. Galil is an elite Israeli spring wheat cultivar obtained from the “Hazera” seed company. It possesses the leaf rust resistance *Lr26* and stripe rust resistance *Yr19* genes but it is susceptible to the leaf and the stripe rust isolates that were used in this research.

**Table 6:** Source of secondary recombinant seeds hybridized to cv. Galil

Primary recombinant lines		Selected BC_1_ (to the HP mutant)			Number of F_1_ seeds (BC_1_ crossed with cv. Galil)^d^
Line designation	Introgression size on chromosome 6B^a^ (cM)	Inoculating pathogen	Infection type^b^	General reaction^c^	
RY-32-3-3	30[7]-107[120]	Leaf rust	0;	HR	44
RL-17-6-10	33[7]-107[120]	Leaf rust	0; −1,1+	R	380
RY-41-1-14	30[7]-107[120]	Leaf rust	1–1+	R	136
RY-32-3-14	33[13]-87	Leaf rust	1–2,2+	MR	33
RY-14-1-7	38–87	Leaf rust	0;-1	R	37
RY-32-3-3	30[7]-107(120]	Stripe rust	0;1=	R	116
RL-17-6-10	33[7]-107[120]	Stripe rust	0;	HR	234
RY-32-3-14	33[13]-87	Stripe rust	0;	HR	362
RY-14-1-7	38–87	Stripe rust	0;1-	R	58

^a^Numbers are based on presence of *Ae. sharonensis* DArT markers or absence of wheat markers. Numbers in square brackets are based on absence of cv. Galil markers in the recombinant line and in CS [23]. RY and RL denote for resistance to stripe rust or to leaf rust, respectively, by which these lines were first selected

^b^Infection types scored according to a 0 to 4 scale. Minus (−) and double minus (=) notations indicate reduced and highly reduced sporulation of uredinia, respectively, compared to classically described infection types

^c^General reactions were as follows: 0 to 0;: highly resistant (HR); 0;1 to 1: resistant (R); 1 to 2: moderately resistant (MR); 2 to 3−: moderately susceptible (MS); and 3− to 4: susceptible (S)

^d^Reflects apparent heterozygous secondary recombinant 6B chromosome

### Pathogens

We used the leaf rust isolate #526–24 and stripe rust isolate #5006 from the stocks of the Institute for Cereal Crops Improvement. The virulence / avirulence (V / Av) formula of these isolates is *Lr1,3,24,26,10,18,21,23,15 / Lr2a,2c,9,16,3 ka,11,17,30* and *Yr6,7,8,9,11,12,17,19,sk,18,A / Yr1,5,10,15,24,26,sp*, for isolates #526–24 and #5006, respectively. Both of these isolates were used to select resistant progenies at the seedling stage and to evaluate adult plant resistance. Both isolates are virulent to cv. Galil and represent highly virulent pathogen races.

### Inoculation and disease evaluation

#### Seedling stage

Seedlings of each generation were tested and selected for susceptibility to leaf and stripe rusts. Plants were grown in small pots in a temperature-controlled greenhouse at 22 ± 2 °C. Seven to 10 days-old seedlings were inoculated by spraying to runoff with ~ 1 mg of urediniospores suspended in 800 μl of lightweight mineral oil Soltrol® 170 Isoparaffin (ChevronPhillips). After evaporation of the oil, the leaf rust-inoculated plants were maintained in a dew chamber at 18 °C for 24 h and then moved to a greenhouse. Stripe rust-inoculated plants were maintained in a dew chamber with 9 °C for 16 h in dark followed by 15 °C in light and then moved to a growth chamber with 15 °C and 12 h light / 12 h dark regime. Symptoms were scored 10–12 days post-inoculation for infection type (IT) on a standard 0–4 scale [46]. ITs of 0–2 were considered indicative of a resistant response, while scores of 3–4 were considered as a susceptible response.

#### Adult plants

##### Leaf rust in the greenhouse

Single homozygous BC_4_F_4_ primary- and BC_2_F_4_ secondary-recombinant plants were grown in 5 L pots placed in a cooled greenhouse and sprayed with urodiniospores at seventh leaf stage (stem elongation, stage 3 - Zadoks scale, [47]). Four groups of plants, each containing two pots for each genotype, were inoculated similarly to the seedling inoculation. Disease level was evaluated after spike emergence, about 3 weeks post inoculation.

##### Stripe rust in the field

Seeds of the same primary and secondary recombinants that were used in seedling assays were planted in nursery plots under field conditions. Each plot (single genotype) consisted of four 1 m rows 20 cm apart and a single margin row 40 cm apart of the spreader cv. Falchetto. The Falchetto plants were inoculated, at seventh leaf stage, with urediniospores six times between January 14 to February 5, by either brushing the upper leaves with a bundle of highly infected leaves, or by dusting the plants with a mixture of urediniospores and talc using a manual air pump. High humidity and cool nights prevailed in the experimental area during this period.

### Shortening the alien segment

Secondary recombinants (SR) with shortened alien segment were obtained by induction of homoeologous recombination in hybrids between the primary recombinants and the wheat cv. Galil, followed by phenotyping and molecular selection as depicted in Fig. 1 and Figure S1. Briefly, selected primary recombinants with different sizes of alien introgression on chromosome 6B (Table 6) were pollinated by the HP mutant and the F_1_ offspring were backcrossed to the HP mutant. Rust resistant plants that were homozygous for *ph1b* were selected and pollinated by cv. Galil. The resulting hybrids were screened for reaction to both pathogens and 6B chromosome constitution of resistant plants was determined using molecular markers (Table 1). Plants with reduced size of the alien segment, compared to the fragment size in parental primary recombinants, were selected and allowed to self-pollinate. Selected rust resistant F_2_ plants were characterized for their *Ph1* genotype. *Ph1*/− were considered secondary recombinants, while homozygous *ph1b* were allowed for another recombination event. Both groups of plants were pollinated by cv. Galil to produce BC_1_ progeny. Chromosome 6B of BC_1_ plants of the latter group was molecularly analyzed and one tertiary recombinant was obtained. All of the rust resistant BC_1_ recombinants were backcrossed again to cv. Galil. BC_2_ plants that were resistant to the leaf and stripe rust isolates were self-pollinated. Resistant BC_2_F_2_ progeny of each recombinant that are also homozygous for the SR 6B chromosome were selected. BC_2_F_3_ seeds of these plants were pooled and used for seed propagation in the greenhouse.

#### Further shortening of the segment

The secondary recombinant (SR) line R-10, in which the alien chromatin extended towards the telomere of the long chromosome arm (right extension; Figure S1, type a) and SR line R-18 in which the alien chromatin extended towards the telomere of the short chromosome arm (left extension; Figure S1, type b) were crossed aiming to curtail the extensions towards the telomeres while maintaining the alien region around the resistance locus (Figure S1, tertiary recombinant). The hybrids were pollinated by cv. Galil and their desired offspring were selected by the molecular markers (Table 1).

### Molecular characterization of the alien segment

#### DNA extraction

Leaves were collected and stored at − 80 °C. Frozen leaf samples (50 mg) were freeze-dried using Lyophilizer (Blue Wave, BW-10-ORD) for 16 h, and grinded for 1 min at 1500 rpm, using two 1/8″ and one 3/16″ stainless steel beads in a Tissue-lyser (GenoGrinder). DNA was extracted using E-Z 96 Plant DNA Kit (Omega) (for PCR analysis), or using DNeasy Plant mini kit (Qiagen) (for GBS), according to manufacturer instructions.

#### Selection of *ph1b* mutants

The presence of the Ph1 allele was detected by marker PSR2120 (No. 10 in Table 1) according to Qu et al. [48]. Plants deficient for the corresponding band were considered as *ph1b*/*ph1b* mutants.

#### Genotyping by sequencing (GBS)

GBS was performed on 100 samples comprised of cv. Galil, the HP mutant, and 74 resistant and 24 susceptible secondary recombinant F_1_ plants (Fig. 1), most of which were derived from the primary recombinant line RY-32-3-14 [23]. DNA was isolated from leaves of young plants (1 month old) as described above. Sequencing was performed according to a modified Restriction site Associated DNA Sequencing (RAD-Seq) method [27] at AgriLife Genomics (Texas). Briefly, genomic DNA was digested with the restriction enzyme PstI and approximately 110 bp were sequenced from both sides of the fragments. For sequencing, single end V4 chemistry with 125 bp kit were used on Illumuna Hiseq 2500 resulting in 40 M reads per sample of cv. Galil and the HP mutant, and 8 M–10 M reads per sample for the rest of the recombinants. Raw data were analyzed by NRGene Ltd. (Ness Ziona, Israel), using the newly assembled wild emmer ‘Zavitan’ genome [25] as a reference.

#### Development of molecular markers for the alien segment

According to the frequency of recombination events, single nucleotide polymorphisms (SNPs) between *Ae. sharonensis* and wheat sequences were mapped along the introgression region. Based on the SNPs, nine PCR primers (markers) were designed following the instructions mentioned at Ayyadevara et al. [49] (No. 1–9 in Table 1). An online available database was used to check for repetitive elements [50].

Conversion from the wild emmer map sequence to CS map sequence and SNPs detection was accomplished as follows: raw reads were de-multiplexed using GBSX [51]. Reads of each genotype were aligned to chromosome 6B of the CS reference genome using Burrows-Wheeler Aligner (BWA) [52]. Samtools package [53] was used to pileup the individual alignment files into one pileup file that was used by bcftools call function [54] to call the SNPs. VariantAnnotation R package [55] was used to read the SNP data into R environment. For each SNP a Fisher Exact test was conducted using a 2X2 contingency table of resistant and susceptible genotypes against the origin of the SNP allele (CS as “reference” SNP or the alien “alternative”). -log *P*-values of the Fisher test were plotted against the position of the corresponding SNP. Higher -log *P*-values (i.e. lower *P*-values) indicated the probability of the SNPs-resistance association to be non-random. SNPs with -log *P* > 16, were assumed to be associated with the resistance locus.

#### Homozygosity of the alien segment

PCR SNP-based primers (markers) were developed to detect the presence/absence of both *Ae. sharonensis* and cv. Galil genotypes (accordingly, presence of cv. Galil marker in a rust resistant plant indicated the heterozygosity of the segment) (No. 11–12 in Table 1).

## Supplementary information

**Additional file 1: Figure S1.** Schematic presentation of the recombinant 6B chromosome from primary to tertiary stages. Primary recombinant contains chromatin of Galil and *Ae.sharonensis* origins. Secondary recombinants present significantly shortened *Ae.sharonensis* chromatin either from the long or the short arm of the chromosome. A cross between the two types of secondary recombinants produced further shortened tertiary recombinant.

**Additional file 2.**

**Additional file 3.**

**Additional file 4.**

## Data Availability

The data that support the findings of this study are available from NRGene, but restrictions apply to the availability of these data, which were used under license for the current study, and so are not publicly available. Data are however available from the authors upon reasonable request and with permission of NRGene.

## Abbreviations

cM: Centimorgan
CS: Chinese Spring
GBS: Genotyping by sequencing
HP: Homoeologous pairing *ph1b* mutant
SNP: Single nucleotide polymorphism
SR: Secondary recombinant
TR: Tertiary recombinant
