# Supplementary material for: Reducing the size of an alien segment carrying leaf rust and stripe rust resistance in wheat
Source: BMC Plant Biol. 2020 Apr 9;20:153. doi: 10.1186/s12870-020-2306-9 (PMC7147030; doi:10.1186/s12870-020-2306-9)
Supplement: Supplementary file 1 — Additional file 1: Figure S1. Schematic presentation of the recombinant 6B chromosome from primary to tertiary stages. Primary recombinant contains chromatin of Galil and Ae.sharonensis origins. Secondary recombinants present significantly shortened Ae.sharonensis chromatin either from the long or the short arm of the chromosome. A cross between the two types of secondary recombinants produced further shortened tertiary recombinant. [file 12870_2020_2306_MOESM1_ESM.zip › Supplementary figure 1_legend.docx]

**Supplementary figure legends**

**Fig. S1.** Schematic presentation of the recombinant 6B chromosome from primary to tertiary stages. Primary recombinant contains chromatin of Galil and *Ae.sharonensis* origins. Secondary recombinants present significantly shortened *Ae.sharonensis* chromatin either from the long or the short arm of the chromosome. A cross between the two types of secondary recombinants produced further shortened tertiary recombinant.
