# Supplementary figures and images for: Reducing the size of an alien segment carrying leaf rust and stripe rust resistance in wheat
Source: BMC Plant Biol. 2020 Apr 9;20:153. doi: 10.1186/s12870-020-2306-9 (PMC7147030; doi:10.1186/s12870-020-2306-9)

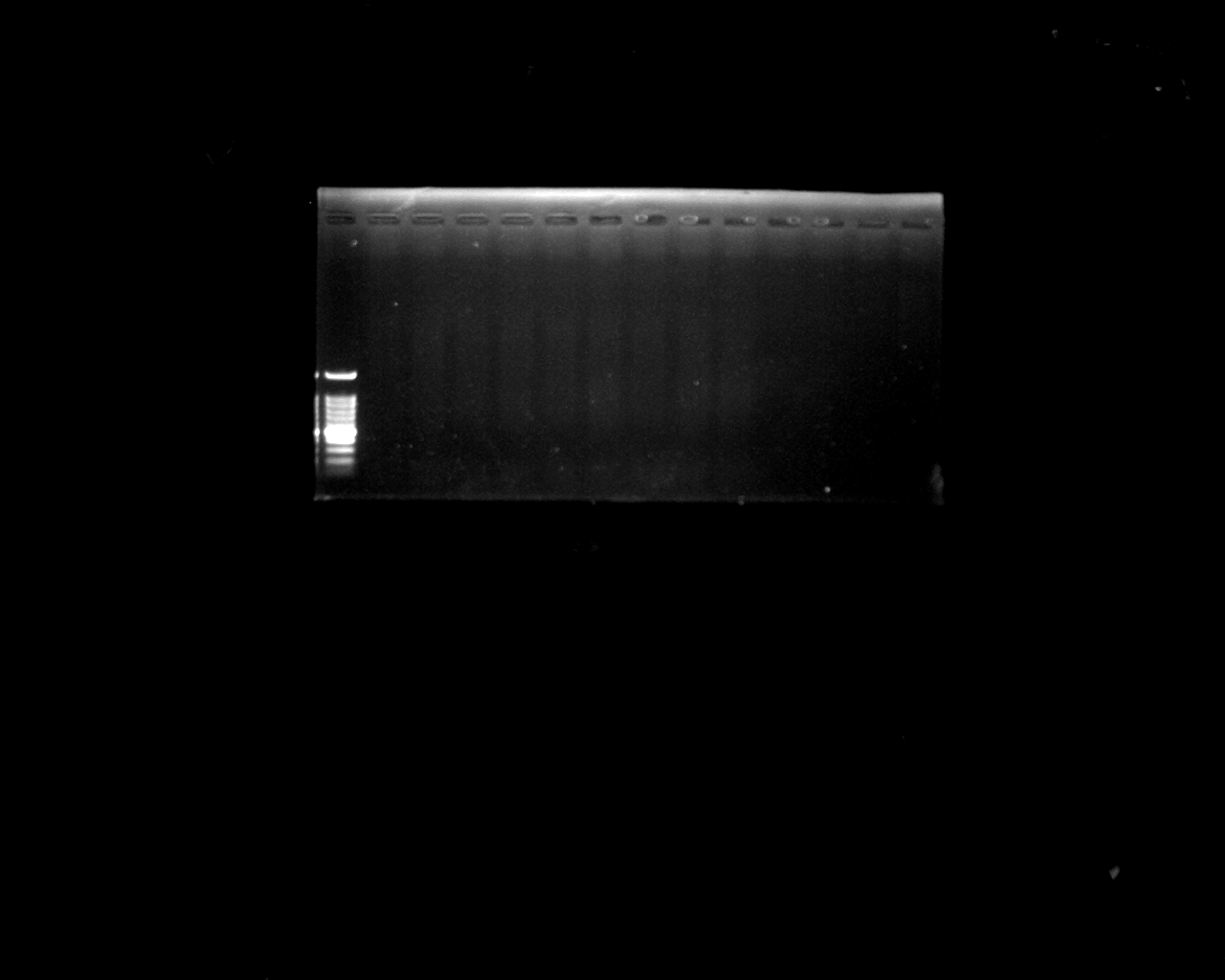

Supplement: Supplementary file 2 — Additional file 2. [file 12870_2020_2306_MOESM2_ESM.tif]

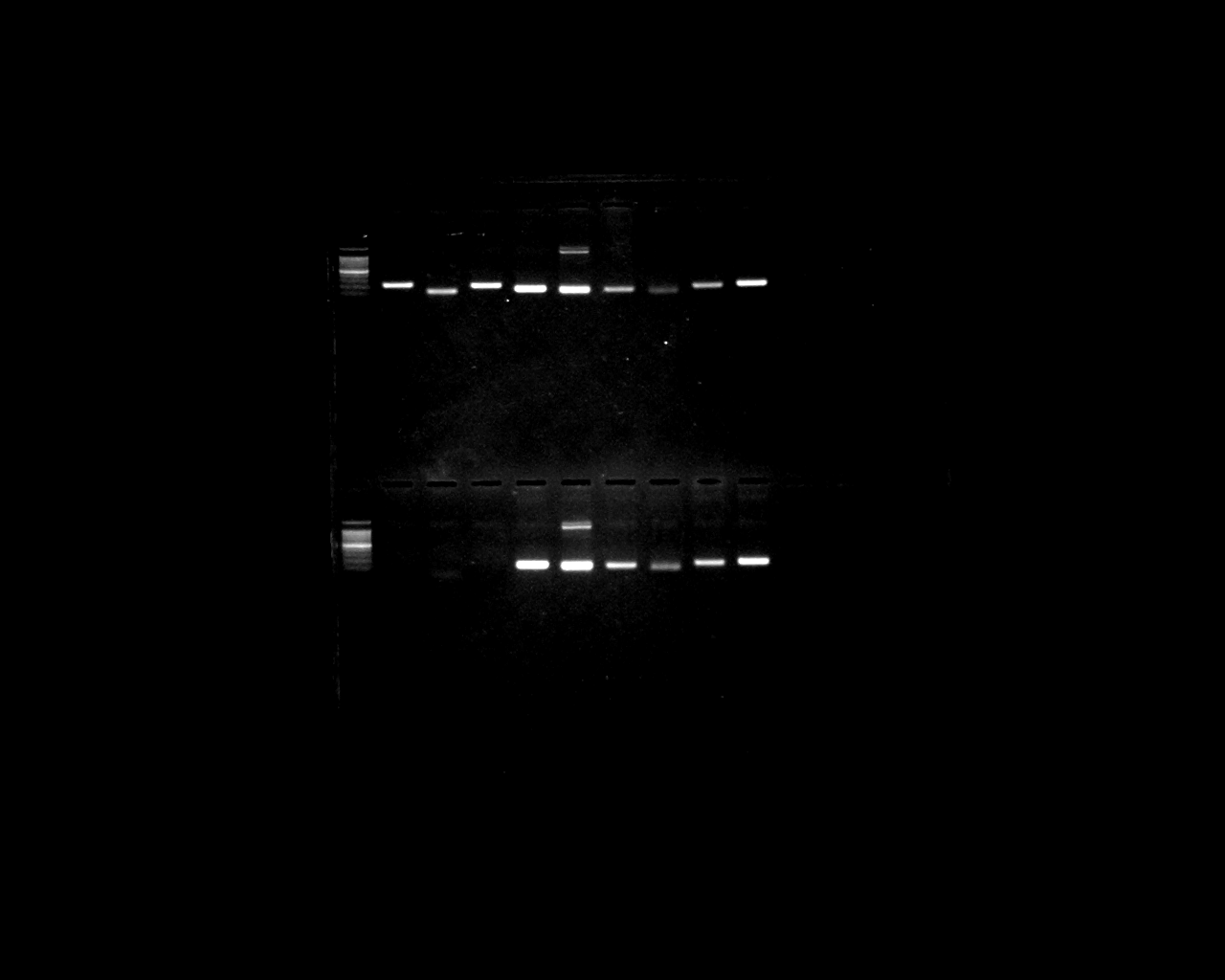

Supplement: Supplementary file 3 — Additional file 3. [file 12870_2020_2306_MOESM3_ESM.tif]

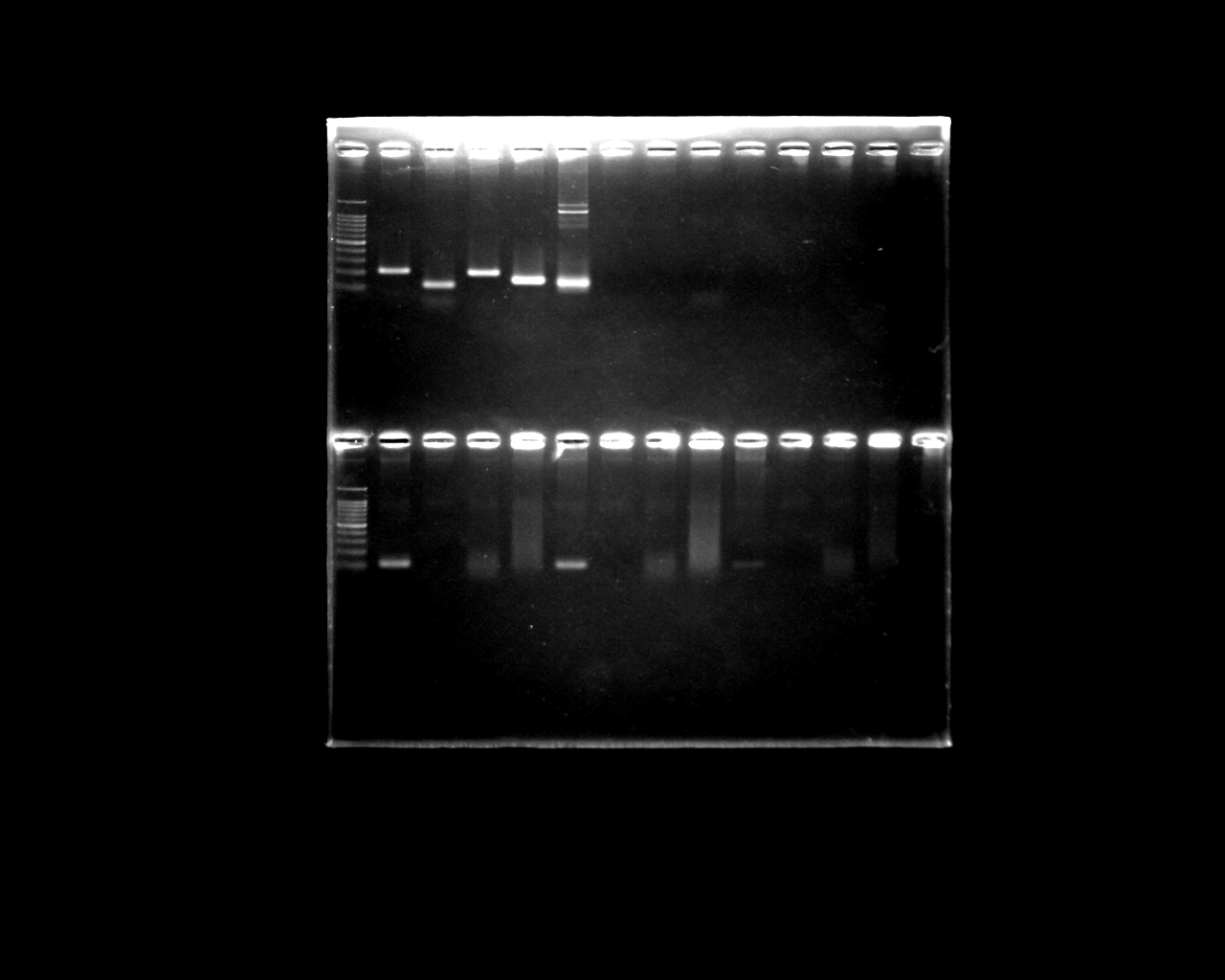

Supplement: Supplementary file 4 — Additional file 4. [file 12870_2020_2306_MOESM4_ESM.tif]
